# Postbiotics as Mitochondrial Modulators in Inflammatory Bowel Disease: Mechanistic Insights and Therapeutic Potential

**DOI:** 10.3390/biom15070954

**Published:** 2025-07-01

**Authors:** Santosh Kumar Prajapati, Dhananjay Yadav, Shweta Katiyar, Shalini Jain, Hariom Yadav

**Affiliations:** 1 USF Center for Microbiome Research, Microbiomes Institute, Morsani College of Medicine, University of South Florida, Tampa, FL 33602, USA; prajapati11@usf.edu (S.K.P.); dhananjay11@usf.edu (D.Y.); jains10@usf.edu (S.J.); 2 Center of Excellence in Aging and Brain Repair, Department of Neurosurgery and Brain Repair, Morsani College of Medicine, University of South Florida, Tampa, FL 33602, USA; 3 Department of Botany, SBN Government PG College, Barwani 451551, India; shwetakatiyar@gmail.com; 4 Department of Internal Medicine, Digestive Diseases and Nutrition, Morsani College of Medicine, University of South Florida, Tampa, FL 33602, USA

**Keywords:** postbiotics, mitochondrial dysfunction, IBD, ROS, oxidative stress

## Abstract

Postbiotics, which are non-viable microbial derivatives including short-chain fatty acids (SCFAs), microbial peptides, and cell wall components, are emerging as novel therapeutic agents for Inflammatory Bowel Disease (IBD). Unlike probiotics, postbiotics offer a safer, more stable alternative while retaining potent bioactivity. IBD, encompassing Crohn’s disease and ulcerative colitis, is characterized by chronic gastrointestinal inflammation, epithelial barrier dysfunction, and immune dysregulation. Recent evidence links mitochondrial dysfunction marked by impaired energy metabolism, oxidative stress, and apoptosis with the pathogenesis and persistence of IBD. Postbiotics have shown the ability to modulate mitochondrial health through multiple mechanisms. SCFAs such as butyrate serve as primary energy substrates for colonocytes, enhancing mitochondrial respiration and promoting biogenesis. They improve mitochondrial function and boost ATP production. Moreover, postbiotics reduce oxidative damage by regulating antioxidant defenses. These antioxidant actions limit epithelial apoptosis and preserve cellular integrity. In addition, postbiotics regulate mitophagy and help maintain mitochondrial quality and reduce inflammation. Structural components such as lipoteichoic acid and peptidoglycan have been shown to interact with mitochondrial pathways and modulate inflammatory responses. Collectively, this review explores the interplay between mitochondrial dysfunction, IBD, and preventive approach using postbiotics. Understanding the connections with postbiotics could open up new avenues for therapeutic interventions aimed at mitigating IBD severity in people with IBD.

## 1. Introduction

In recent years, there has been growing interest in the therapeutic potential of microbiome-derived metabolites in maintaining gastrointestinal health and managing chronic intestinal disorders. Among these, the byproducts or structural components of non-viable probiotics (postbiotics) have garnered significant attention [1].

Unlike probiotics, postbiotics are safer and more stable, with minimal risk of microbial translocation or adverse effects in immunocompromised individuals [2]. Postbiotics include a diverse range of compounds such as short-chain fatty acids (SCFAs), microbial peptides, cell wall fragments (like lipoteichoic acids), extracellular polysaccharides, and enzymes [2]. These bioactive molecules are produced during the fermentation of dietary substrates by commensal gut microbiota and are increasingly recognized as important modulators of host physiology.

Among all postbiotics, SCFAs, particularly butyrate, acetate, and propionate, are the most widely studied and exhibit potent anti-inflammatory, immunomodulatory, and metabolic regulatory effects [3]. These molecules are especially critical for maintaining the homeostasis of the intestinal epithelium, which acts as a barrier between the host and the external environment. Butyrate is the primary energy source for colonocytes and is essential for epithelial cell proliferation, differentiation, and maintenance of tight junction integrity [4]. Importantly, butyrate also influences gene expression, oxidative stress responses, and immune function via epigenetic modulation and signaling through G-protein coupled receptors (GPCRs) and histone deacetylase (HDAC) inhibition [4].

Postbiotics have been successfully explored in a range of inflammatory disorders, including respiratory infections, dermatitis, metabolic syndrome, and particularly inflammatory bowel disease (IBD) [5]. IBD, which encompasses Crohn’s disease (CD) and ulcerative colitis (UC), is a chronic relapsing-remitting inflammatory condition of the gastrointestinal tract [6,7]. Its pathogenesis is complex, involving genetic susceptibility, environmental factors, immune dysregulation, and gut microbiota disturbances. Multiple studies have documented a significant reduction in butyrate-producing bacterial populations, such as *Faecalibacterium prausnitzii* and *Roseburia* spp. in IBD patients [8,9]. This dysbiosis results in a decline in SCFA levels and contributes to the disruption of the mucosal barrier, excessive immune activation, and oxidative damage, all of which are hallmarks of IBD pathophysiology [6,7]. IBD is characterized by chronic inflammation due to the release of inflammatory markers such as interleukin (IL-1β), IL-6 and tumor necrosis factor-alpha (TNF-α), mucosal injury, and immunological dysregulation, all of which cause debilitating symptoms and systemic effects [10] often associated with gut dysbiosis. Gut dysbiosis is described as a reduced microbial diversity along with a decrease in healthy bacterial species [11]. In healthy individuals, immune tolerance toward commensal microbiota is essential for maintaining intestinal immune homeostasis. Dysregulation of this balance can result in inappropriate immune activation, contributing to the development and progression of IBD [12]. Some of the most convincing evidence comes from germ-free mouse models, which develop chronic intestinal inflammation after colonization with commensal gut bacteria but are disease-free in bacteria-free conditions, implying that non-pathogenic enteric bacteria play a primary role in the pathogenesis of IBD [13,14]. Furthermore, some studies suggest that using “beneficial bacteria” or probiotics helps improve IBD [15]. However, the exact mechanism is yet to be known. Emerging evidence demonstrated that mitochondrial dysfunction is one of the contributing factors between gut dysbiosis and the development of IBD [16,17,18]. Mitochondria are the organelles responsible for energy production and cellular balance, which are critical for gastrointestinal health. A decline in mitochondrial activity has been observed in inflammatory diseases such as CD and UC due to increasing oxidative damage and altered dynamics [19]. Further, research has demonstrated a bidirectional link between mitochondria and gut microbiota in inflammatory disorders [20]. Thus, the dysregulation of these pathways impairs mitochondrial energy production and antioxidant defense, leading to epithelial barrier dysfunction, increased oxidative stress, and immune activation. Furthermore, the gut microbiota regulates transcription factors and enzymes involved in mitochondrial biogenesis, including Peroxisome Proliferator-Activated Receptor Gamma Coactivator 1-Alpha (PGC-1α), Sirtuin 1 (SIRT1), and AMP-Activated Protein Kinase (AMPK) genes [21]. The gut microbiota and its metabolites, including SCFA and secondary bile acids, have a role in energy production, reactive oxygen species (ROS) regulation, and inflammation in the gut by inhibiting (TNF-α)-mediated immune responses and inflammasomes like NOD-like Receptor Family Pyrin Domain Containing 3 (NLRP3) [21,22]. On the other hand, mitochondria, particularly mitochondrial ROS generation, play an important role in regulating the gut microbiota via altering intestinal barrier function and mucosal immune responses [21].

Therefore, there is a correlation among IBD, gut dysbiosis, and mitochondrial dysfunction. Given their low immunogenicity, ease of formulation, and broad-spectrum health benefits, postbiotics are becoming attractive candidates for therapeutic development in chronic diseases such as IBD [23]. Postbiotics possess the ability to interact with host mitochondria, functioning as key regulators of epithelial inflammation. This interaction provides a compelling mechanistic basis for its therapeutic application, particularly in conditions like gut dysbiosis including IBD, where mitochondrial signaling plays a critical role in maintaining intestinal homeostasis. Thus, this review explores the current evidence linking postbiotics to IBD with a special emphasis on the mitochondrial mechanisms that underpin their effects. By highlighting both preclinical findings and clinical applications, this work aims to provide an integrated perspective on the potential of postbiotics as mitochondrial modulators in the prevention and treatment of IBD.

## 2. Mitochondrial Dysfunction in IBD

### 2.1. Oxidative Stress in IBD

Mitochondria are double-membraned organelles with circular DNA that regulate energy production, apoptosis, immune responses, and epithelial integrity. In IBD, mitochondrial dysfunction has been closely linked to chronic inflammation, impaired epithelial barrier function, and increased intestinal permeability [24,25]. When mitochondrial activity is compromised, cell experience decreased ATP production, excessive ROS generation, and altered metabolic signaling, all of which contributed to the pathogenesis of IBD [24,25,26].

Oxidative stress is one of the central contributors to intestinal inflammation in IBD and is tightly linked with mitochondrial dysfunction. Reactive oxygen and nitrogen species (ROS/RNS) can damage DNA, proteins, and lipids, impairing cellular function and promoting apoptosis [27]. Elevated levels of ROS/RNS have been reported in both IBD patients and experimental colitis models [28,29], along with oxidized molecules in saliva, plasma, and breath [30]. Further, studies have also shown reduced antioxidant levels, such as glutathione and vitamins C and E, in IBD patients [31]. Mice deficient in glutathione peroxidase develop spontaneous colitis [32], and antioxidant treatment restores glutathione in DSS-induced colitis models [33].

In-depth studies have revealed increased DNA and lipid oxidation in IBD through mtDNA damage, further highlighting oxidative stress as a key factor contributing to the pathogenesis of IBD [34,35]. In the context of IBD, these polymorphisms influence mitochondrial number, redox balance, and ROS production, thereby exacerbating epithelial stress and inflammation [36]. These mutations tend to accumulate during the heightened epithelial regeneration process seen in chronic inflammation, particularly with aging, potentially overwhelming replication mechanisms and perpetuating further genetic instability [37]. For example, prohibitin, a mitochondrial chaperone protein, plays a crucial role in maintaining mitochondrial integrity. Theiss et al. reported reduced prohibitin expression in both animal models of colitis and IBD patients [38]. In vitro studies with Caco2-BBE human intestinal epithelial cells showed that prohibitin overexpression protected against oxidative stress-induced barrier dysfunction, while its loss triggered increased permeability and inflammation [38]. Similarly, Jackson et al. demonstrated that prohibitin 1, a key inner mitochondrial membrane protein essential for proper respiratory chain assembly and function, is significantly reduced during IBD [39]. This reduction impairs mitochondrial function, contributing to epithelial cell stress and inflammation. Importantly, their study further revealed that Paneth cells, specialized epithelial cells vital for maintaining gut microbial balance and mucosal immunity, are particularly vulnerable to mitochondrial dysfunction. The loss of mitochondrial integrity in these cells was shown to contribute to the development of ileitis in murine models [39]. These findings carry significant translational implications, especially for a subset of CD patients who exhibit intrinsic Paneth cell defects, linking mitochondrial dysfunction directly to disease pathogenesis in these individuals [39].

Additionally, mitophagy is another vital mitochondrial quality control mechanism, triggered under conditions of excessive stress such as the accumulation of misfolded proteins or mitochondrial depolarization. It activates the PINK1/Parkin pathway to eliminate damaged mitochondria [40,41]. In murine models of colitis, such as DSS-induced colitis, PGC-1α, a regulator of mitochondrial biogenesis, is upregulated following mitophagy to restore mitochondrial numbers and function [42]. However, excessive mitochondrial fission and impaired fusion are common in inflamed intestinal epithelium, and the inhibition of mitochondrial fragmentation has been shown to alleviate colitis severity [43]. The disruption of mitochondrial homeostasis between fission and fusion in IBD is primarily attributed to altered cellular energy regulation. Accordingly, the following section explores the interplay between energy metabolism and intestinal barrier integrity in the context of IBD.

### 2.2. Mitochondrial Energy Metabolism and Barrier Integrity in IBD

Mitochondria are pivotal for maintaining energy homeostasis in the intestinal epithelium, especially under the high metabolic demands during IBD. Evidence indicates that the intestinal mucosa of IBD patients exists in an energy-deficient state, characterized by reduced ATP levels and diminished energy charge potential, implicating mitochondrial dysfunction in disease pathology [44]. Key energy-dependent processes—including β-oxidation, maintenance of tight junctions (TJs), and epithelial barrier integrity—are notably impaired in both human and animal IBD models [44].

Intestinal epithelial cells (IECs) act as a selective barrier against luminal antigens and maintain nutrient absorption. Aberrant expression of genes regulating IEC differentiation is observed under inflammatory conditions, affecting mucosal defense [45]. For instance, patients with UC display a reduction in goblet cells and mucus production [46]. Similarly, Muc2, the primary mucin component of the intestinal mucus layer, is essential for barrier protection, and its deficiency in mice leads to spontaneous colitis due to increased epithelial exposure to luminal microbes [47]. A report shows that Muc2-deficient mice spontaneously develop colitis, underlining the importance of goblet cells in barrier function [48]. Paneth cells also show dysfunction associated with IBD susceptibility genes, including *NOD2*, *ATG16L1*, and *XBP1*, which are linked to decreased α-defensin secretion and spontaneous CD-like ileitis in knockout mouse models [49,50]. In IBD, tight junction (TJ) proteins such as occludin, claudin-1, and zonula occludens-1 (ZO-1), which regulate paracellular permeability, are energy-dependent structures that become degraded during mitochondrial impairment, leading to increased intestinal permeability and barrier dysfunction [51]. Mitochondrial damage caused by external insults like nonsteroidal anti-inflammatory drugs exposure compromises TJ integrity and increases gut permeability [52,53]. In CD, immune reactivity to gut microbiota has been linked to barrier dysfunction [54], although the underlying role of metabolic stress and mitochondrial dysfunction requires further elucidation.

β-Oxidation, particularly the oxidation of butyrate, a primary fuel for colonic epithelial cells, is also impaired in IBD. Reduced butyrate metabolism is evident in both IBD patients and colitis animal models [55]. Inhibition of butyrate oxidation triggers mucosal inflammation in mice [56], while UC patients exhibit decreased mitochondrial acetoacetyl CoA thiolase, likely due to increased mitochondrial ROS [57]. Moreover, polymorphisms in *SLC22A5*, encoding the carnitine transporter OCTN2, are a genetic risk factor for IBD, and its deletion in mice results in colitis [58,59]. Thus, mitochondrial impairment in β-oxidation not only affects energy balance but also compromises epithelial integrity and immune tolerance.

### 2.3. Apoptosis and Cell Death Pathways in IBD Pathogenesis

Apoptosis of IECs is a central pathological feature in IBD, with studies consistently reporting elevated IEC apoptosis that correlates with inflammation severity [60,61]. The extrinsic apoptotic pathway is prominently involved, driven by death receptors such as CD95 (Fas), TNFR1, and TRAIL [62,63]. In IBD patients, an aberrant upregulation of Fas ligand (CD95L), BAX, and p53 has been observed in IECs—molecules typically restricted to immune and Paneth cells in healthy individuals [64]. Mitochondria-mediated apoptosis in IECs is primarily governed by p53 and its downstream target, PUMA (p53-upregulated modulator of apoptosis), which represses anti-apoptotic Bcl-2 proteins [64]. Both p53 and PUMA are significantly increased in human UC and DSS-induced colitis in mice, particularly within the lower crypts [65]. Genetic deletion of p53 or PUMA reduces IEC apoptosis but does not attenuate inflammation, suggesting a p53-independent mechanism for PUMA-driven apoptosis regulated by Chk1 kinase [65].

TNF-α, an inflammatory cytokine in IBD, promotes IEC death under conditions of impaired NF-κB signaling. TNF-α does not induce apoptosis in healthy IECs; however, disruption of survival pathways particularly through NEMO (NF-κB essential modulator) deletion renders IECs susceptible to TNF-RIPK1-dependent apoptosis [64]. Prolonged NF-κB activation following TNF-α stimulation exacerbates mucosal damage, which can be mitigated by RIPK1 inhibitors that prevent apoptosis without compromising survival signaling [64]. Additionally, A20 (TNFAIP3), a negative regulator of NF-κB, is overexpressed in IBD patient IECs and modulates RIPK1-mediated apoptosis; inhibition of RIPK1 protects A20-overexpressing mice from TNF-induced cell death [66,67]. TAK1, another upstream regulator of NF-κB, also controls apoptosis by promoting FLIP degradation and the formation of the RIPK1–FADD–caspase–8 complex, pushing IECs toward apoptotic rather than necroptotic death [68].

MicroRNAs also contribute to apoptosis regulation in IBD. miR-665 is upregulated in both IBD patients and DSS colitis models, promoting IEC apoptosis, whereas miR-665 inhibition alleviates disease severity [60]. In CD, reduced expression of pro-apoptotic BAX in the lamina propria and elevated Bcl-2 in mesenteric adipose tissue (MAT) reflect impaired immune cell apoptosis and resistance to steroid therapy. Additionally, increased caspase-3 activity in MAT supports a complex regional regulation of apoptosis [64].

Sam68, a multifunctional RNA-binding protein involved in RNA splicing, signal transduction, and NF-κB-mediated inflammatory responses, is markedly increased in IECs from UC patients [69]. In DSS colitis models, Sam68 gene overexpression correlates with elevated apoptotic markers and NF-κB signaling, further supporting its role as a regulator of IEC death during inflammation [64].

Together, these findings highlight the complex interplay between death receptor signaling, mitochondrial dysfunction, immune regulation, and epigenetic factors in mediating IEC apoptosis, a process central to barrier dysfunction and disease progression in IBD.

## 3. Microbiota–Mitochondria Crosstalk in IBD Pathogenesis

The interplay between gut microbiota and host mitochondria has emerged as a crucial factor in the pathogenesis of IBD. Increased diversity of microbial metabolites, particularly SCFAs like butyrate, plays a protective role by enhancing mitochondrial function and regulating epithelial cell energy metabolism and apoptosis [56,70]. In IBD, dysbiosis leads to a depletion of butyrate-producing bacteria such as *Faecalibacterium prausnitzii*, resulting in impaired mitochondrial oxidative phosphorylation and increased vulnerability to inflammation [3,55]. Further, mitochondrial dysfunction in IECs can exacerbate dysbiosis by altering oxygen gradients in the gut, favoring the overgrowth of facultative anaerobes like *Escherichia coli*, commonly observed in IBD [71]. Invasive *Escherichia coli* strains, commonly associated with CD, reduce ΔΨm and ATP production while inducing DNM1L-mediated mitochondrial fission and cytochrome c release, triggering apoptosis in IECs [72]. These bacteria also suppress mitochondrial fusion and biogenesis genes such as optic atrophy 1 (OPA1) and PGC-1α, impairing mitochondrial recovery even after DNM1L inhibition [44,72,73]. Colonic acid, a key biofilm component produced by *Escherichia coli*, promotes mitochondrial fragmentation in intestinal cells through a dynamin-related protein 1 (DRP1)-dependent mechanism [74]. Additionally, it enhances the unfolded protein response (UPR) by activating the stress-responsive transcription factor ATFS-1 in the context of mitochondrial stress [74]. ATFS-1 is a transcription factor regulating the mitochondrial unfolded protein response in *Caenorhabditis elegans*, with ATF5 serving as its functional counterpart in mammals [75]. Furthermore, a group of bacterial metabolites including betaine, methionine, and homocysteine initiates a signaling cascade involving the nuclear receptor 5A, which subsequently activates Hedgehog signaling to modulate the mitochondrial fission–fusion balance in intestinal cells [76].

Moreover, a recent study demonstrated that defects in autophagy-related genes such as *ATG16L1* impair mitophagy, leading to the accumulation of dysfunctional mitochondria and further amplifying intestinal inflammation [64]. Collectively, these findings highlight a bidirectional and pathogenic loop where gut microbial imbalance and mitochondrial dysfunction reinforce each other, contributing to the chronic inflammatory state in IBD.

Another significant microbial alteration in IBD is the expansion of sulfate-reducing bacteria (SRB) [77]. Metagenomic sequencing reveals a three-fold increase in SRB, including *Desulfovibrio piger* and *Bilophila wadsworthia* in active UC, correlating with decreased mitochondrial Complex IV activity in the colonic epithelium [77]. Hydrogen sulfide (H_2_S) levels in colonic gas samples from UC patients are 2–4 times higher than controls, associated with elevated serum cytochrome c and signs of mitochondrial-dependent apoptosis [78]. The mechanistic basis for these observations lies in the fact that H_2_S produced by *Desulfovibrio* species competitively inhibits cytochrome c oxidase (Complex IV) at concentrations > 0.2 mM, disrupting the electron transport chain and reducing ATP synthesis [79]. Additionally, *Bilophila wadsworthia*-mediated taurine metabolism generates hydrogen sulfide that damages mitochondrial DNA and impairs mitochondrial autophagy through inhibition of the ULK1 complex [80].

Tryptophan metabolites represent another class of microbiome-derived compounds with a beneficial effect on mitochondria. Indole 3-propionic acid (IPA) is a tryptophan deamination product derived from *Lactobacillus reuteri*, *Akkermansia*, and *Clostridium* genus, which mediate intracellular signaling activity [81]. IPA alleviates DSS-induced colitis in mice by modulating macrophage glycolipid metabolism, thereby reducing intestinal inflammation and promoting mucosal healing [82]. The bacterial metabolite indole strengthens tight junction integrity in intestinal epithelial cells and reduces markers of inflammation, thereby enhancing gut barrier function [83]. Indole-3-aldehyde levels are significantly reduced in stool samples from IBD patients, correlating with impaired mitochondrial respiration in intestinal biopsies as measured by Seahorse analysis [84]. IPA prevents TNF-α-induced mitochondrial ROS production by upregulating mitochondrial antioxidant enzymes through Nrf2 activation [85]. Thus, evidence highlights IPA as a prominent regulator of mitochondrial biogenesis in IBD pathogenesis. Targeting these pathways holds therapeutic potential.

**Figure 1:**
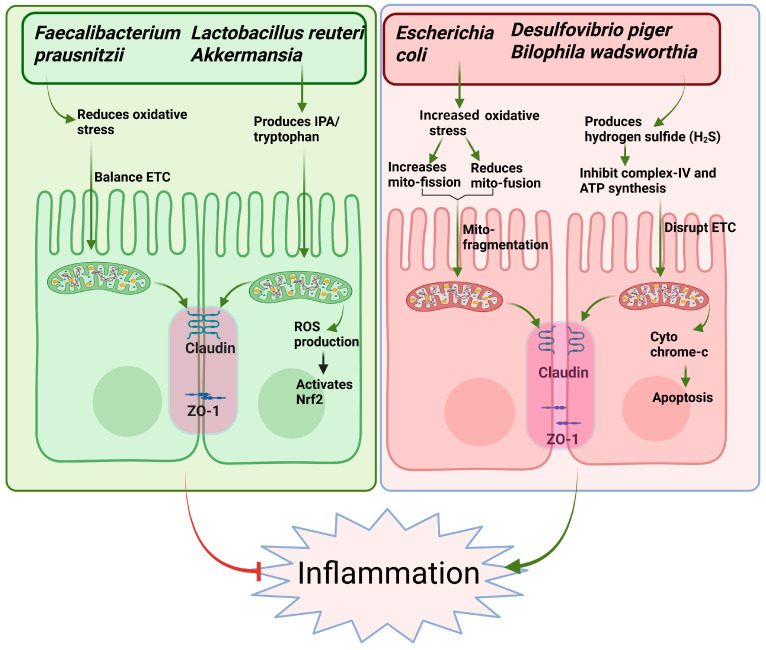
Differential effects of gut microbial species on mitochondrial homeostasis and intestinal inflammation in IBD. Right side (green) represents beneficial microbes such as *Faecalibacterium prausnitzii*, *Lactobacillus reuteri*, and *Akkermansia* that enhance mitochondrial function by reducing oxidative stress, balancing the electron transport chain (ETC), producing indole propionic acid (IPA) and tryptophan, reduced ROS production and activating antioxidant pathways like Nrf2. These effects promote tight junction integrity (Claudin, ZO-1) and suppress inflammation. In contrast, the left side (red) shows that pathogenic microbes, including *Escherichia coli*, *Desulfovibrio piger*, and *Bilophila wadsworthia* disrupt mitochondrial dynamics by increasing mitochondrial fission, reducing fusion, producing hydrogen sulfide (H_2_S), and inhibiting complex IV and ATP synthesis. These disruptions lead to ETC impairment, cytochrome c release, apoptosis, tight junction damage, and heightened intestinal inflammation.

Trimethylamine-N-oxide (TMAO), another metabolite produced through microbial metabolism of choline, carnitine, and phosphatidylcholine by gut bacteria such as *Escherichia coli* and *Clostridium* spp. [86], has been implicated in promoting mitochondrial dysfunction and inflammation [87]. It is reported that TMAO-induced activation of the innate immune system is closely involved in IBD pathogenesis [88]. TMAO induces oxidative stress and activates NLRP3 inflammasome in fetal human colon cells, indicating that TMAO can trigger the activation of the NLRP3 inflammasome and lead to inflammatory responses in the intestinal endothelium [88]. Chaochi et al. demonstrated the involvement of TMAO in the pathogenesis of IBD. They found that TMAO significantly inhibits the expression of ATG16L1, LC3-II, and p62 and triggers NLRP3-activated inflammasome with the production of ROS in human colon cells, contributing to the development of intestinal wall damage [88]. These findings suggest that modulation of TMAO and its microbial producers may offer novel therapeutic avenues for IBD via targeting mitochondrial dysfunction (Figure 1).

## 4. Postbiotics in Inflammatory Bowel Disease: Insights from Preclinical and Clinical Studies

The beneficial effects of postbiotics in the context of IBD have been extensively explored in both preclinical animal models and clinical trials. Postbiotics such as SCFAs, butyrate, and heat-inactivated *Lactobacillus paracasei* (D3-5) ameliorates leaky gut, inflammation and improves physical and cognitive functions [89]. In preclinical studies, the administration of butyrate and other SCFAs has consistently demonstrated protective effects against chemically induced colitis, including DSS (dextran sulfate sodium)- and TNBS (2,4,6-trinitrobenzenesulfonic acid)-induced models [90,91]. These interventions led to reductions in inflammatory cytokines such as TNF-α, IL-1β, and IL-6 while promoting anti-inflammatory cytokines like IL-10. Moreover, butyrate administration improved histological scores, reduced mucosal damage, and promoted epithelial regeneration [92]. For example, in a DSS-induced colitis model, oral administration of sodium butyrate significantly ameliorated disease activity index, preserved colonic length, and restored goblet cell numbers [93,94]. These outcomes were associated with enhanced tight junction protein expression, including occludin and claudin-1, suggesting a restoration of epithelial barrier integrity [94].

Beyond SCFAs, bacterial lysates and inactivated probiotics have also shown promise. A study by Chorawala et al. revealed that cell wall components derived from *Lactobacillus casei, Lactobacillus acidophilus,* and *Lactobacillus rhamnosus* have significant protective effects against LPS-induced colitis in rats [95]. These components reduce the expression of key pro-inflammatory cytokines, including TNF-α, IL-6, and IL-1β, and simultaneously elevate the anti-inflammatory cytokine IL-10, indicating strong immunomodulatory activity [95]. Additionally, oxidative stress was markedly attenuated, as shown by decreased levels of malondialdehyde (MDA) and myeloperoxidase (MPO), and increased activities of antioxidant enzymes superoxide dismutase (SOD) and catalase (CAT). Histopathological and hematological analyses revealed improved tissue integrity and reduced leukocytosis, with the most pronounced effects observed from *Lactobacillus acidophilus* cell wall extract [95].

A study using lysate of *Lactobacillus plantarum* K8 effectively reduced colonic inflammation in DSS-induced colitis by lowering pro-inflammatory cytokines TNF-α and IL-6 [96]. It improved mucosal integrity and reduced epithelial damage, edema, and neutrophil infiltration. High-dose lysate significantly increased TLR-2 mRNA expression, supporting epithelial defense mechanisms [96]. Likewise, heat-killed *Bifidobacterium breve* improved DSS-induced colitis and inflammation in rodent models [97]. Moreover, the heat-killed body of *Lactobacillus brevis* SBC8803 ameliorates intestinal injury in a murine model of colitis by enhancing the intestinal barrier function [98].

### Clinical Studies on Postbiotics in IBD Patients: Evidence of Translational Impact

Although much of the mechanistic understanding of postbiotics has emerged from in vitro and animal studies, an increasing number of clinical investigations have begun to demonstrate their therapeutic potential in human IBD populations. These studies provide a translational bridge between preclinical findings and real-world applications.

Lührs et al. reported that rectal administration of butyrate in UC patients led to improved clinical outcomes and significantly inhibited NF-κB activation in colonic lamina propria macrophages, indicating a reduction in inflammation at the mucosal level [99]. Similarly, Segain et al. demonstrated that oral sodium butyrate reduced pro-inflammatory cytokine expression in CD patients by inhibiting NF-κB, suggesting a conserved anti-inflammatory mechanism across IBD subtypes [100].

In a randomized controlled trial, Jin et al. showed that *Saccharomyces boulardii*-derived postbiotics ameliorated UC symptoms and corrected gut microbial dysbiosis, particularly by restoring the Firmicutes/Bacteroidota (F/B) ratio and increasing *Akkermansia* and *Dubosiella* abundance [101]. Furthermore, Enck et al. administered microbial lysates of *Escherichia coli* and *Enterococcus faecalis* to UC patients, resulting in reduced relapse rates and improved mucosal recovery [102].

A recent multicenter double-blind study using heat-inactivated *Bifidobacterium bifidum* MIMBb75 also showed significant symptom relief in patients with irritable bowel syndrome, a condition often overlapping with IBD in symptomatology and gut barrier dysfunction [103]. Although not conducted in IBD patients per se, this trial supports the safety and efficacy of postbiotics in modulating gut function.

Taken together, these clinical findings suggest that postbiotics, particularly SCFAs, microbial lysates, and heat-inactivated probiotics, can favorably modulate intestinal inflammation, barrier function, and microbiota composition in humans. Continued investigation through well-powered clinical trials is essential to solidify their role as adjunct or standalone therapeutics in IBD management (Table 1).

Moreover, Huang et al. demonstrated that combining butyrate with active vitamin D significantly reduced *Salmonella* colitis severity in C57BL/6 mice [112]. This combination downregulated pro-inflammatory cytokines IL-6, IL-8, TNF-α, and mIL-1β. The synergistic effect was mediated via activation of the vitamin D receptor (VDR).

## 5. Postbiotics in Mitochondrial Dysfunction and IBD Therapy

Postbiotics like butyrate directly impact mitochondrial health. Butyrate enters colonocytes via monocarboxylate transporters (MCTs) and serves as a primary fuel source, enhancing mitochondrial respiration [113]. It activates the AMPK-PGC1α-NRF1-TFAM axis, promoting mitochondrial biogenesis and restoring bioenergetic function [114]. In mouse models of colitis, butyrate supplementation has been shown to normalize mitochondrial membrane potential, restore cristae architecture, and elevate ATP levels [115]. Another major mitochondrial mechanism involves oxidative stress regulation. IBD mucosa exhibits high levels of ROS and mitochondrial lipid peroxidation, contributing to DNA and protein damage [116]. Postbiotics enhance antioxidant defenses by upregulating SOD2 and glutathione synthesis while downregulating NADPH oxidase activity [117]. This redox regulation reduces epithelial apoptosis and promotes survival under inflammatory stress. Postbiotics derived from *Lactobacillus brevis* and *Lactobacillus casei* modulate the mitochondrial (intrinsic) apoptosis pathway by influencing Bcl-2 family proteins, upregulating anti-apoptotic Bcl-2 and downregulating pro-apoptotic Bax [118]. This shift inhibits caspase-3 activation and prevents epithelial cell loss of cells. Furthermore, postbiotics promote mitophagy, the selective autophagic clearance of damaged mitochondria [119]. By enhancing PINK1/Parkin signaling and increasing autophagosome formation, postbiotics help maintain a healthy mitochondrial pool, critical for intestinal homeostasis [119]. Collectively, these mechanisms support the concept that postbiotics act not merely as metabolic supplements but as mitochondrial therapeutics, modulating core organelle functions to restore gut health in IBD.

Postbiotics possess several mitochondrial-linked properties like antioxidant, mitochondrial biogenesis, and apoptotic pathways in IBD. Postbiotics frequently have antioxidant qualities that can scavenge excessive ROS and preserve the integrity of mitochondria. For example, metabolites butyrate, a SCFA which has been shown to lower oxidative stress, stabilizes mitochondrial membranes and encourages effective ATP synthesis. Postbiotics promote mitochondrial biogenesis by triggering signaling pathways like the PGC-1α pathway. Postbiotics such as butyrate, lactate, and propionate enhance mitochondrial function by modifying energy metabolism and lowering ROS generation [120]. Their involvement in the gut microbiome and preserving epithelial barriers shows the possible approaches in the treatment of IBD [120].

It has been reported that postbiotics Inhibit the pro-inflammatory signaling pathways, such as NF-kb, which are steadily upregulated during impaired mitochondrial function and oxidative stress. Postbiotics may indirectly promote mitochondrial health by reducing cellular damage and maintaining tissue integrity through their ability to reduce inflammation. Additionally, postbiotics can regulate mitochondrial-dependent apoptosis. Mitochondrial dysfunction can cause excessive apoptosis, which can lead to tissue damage and intestinal epithelial cell loss. Postbiotics that encourage balanced cell death may contribute to the preservation of intestinal epithelial integrity, which is crucial for the treatment of IBD [23].

The potential of bacterial cell wall constituents, lipoteichoic acid (LA), and peptidoglycan to interact with the mitochondrial signaling pathway is studied by numerous researchers [121,122]. LA and peptidoglycans can stimulate immune responses in addition to regulating metabolic processes in mitochondria, offering an alternate strategy to treating malfunctioning mitochondria in inflammatory conditions such as IBD. Further, gut bacteria-secreted proteins, for example, from the species of *Lactobacillus* and *Bifidobacterium*, could exert beneficial effects on mitochondrial function by affecting the pathways of metabolism and inflammation. The mechanism involved is secretion of specific proteins by gut bacteria such as p75 and p40 from *Lactobacillus rhamnosus GG* has been shown to enhance mitochondrial function by activating the PI3K/Akt pathway and reducing mitochondrial apoptosis in intestinal epithelial cells [123,124]. Similarly, the BopA protein from *Bifidobacterium bifidum* promotes epithelial cell adhesion and modulates host immune responses, potentially contributing to mitochondrial protection under inflammatory stress [125]. Furthermore, heat-killed *Bifidobacterium bifidum* B1628 alleviates dextran sulfate sodium-induced colitis in mice through gut microbiota modulation [105]. Similarly, postbiotics from heat-treated *Bifidobacterium longum* CECT-7347 exhibit anti-inflammatory and antioxidant effects both in vitro and in vivo, marked by increased IL-10 expression and enhanced free radical scavenging [126]. These findings suggest a mitochondrial-dependent mechanism in the regulation of IBD. Moreover, *Limosilactobacillus fermentum HF06*-derived paraprobiotic (6-PA) and postbiotic (6-PS) alleviate DSS-induced UC by reducing inflammation, oxidative stress, and fecal moisture while preserving colon length and body weight. They enhance gut barrier integrity via upregulation of TJPs like ZO-1 and occludin and restore microbial balance by modulating key bacterial taxa such as *Bifidobacterium*, *Faecalibaculum*, *Muribaculaceae*, *Corynebacterium,* and SCFA levels [107]. Moreover, Pradhan et el. demonstrated that lipoteichoic acid (LTA) derived from *lactobacilli* exhibits anti-inflammatory and protective effects against colitis. In LPS-stimulated HT-29 cells, LTA modestly modulated cytokine levels by slightly increasing IL-10 and decreasing TNF-α. Furthermore, LTA administration in colitis-induced mice significantly improves gut permeability, myeloperoxidase activity, and histological damage [108].

This double-blind placebo-controlled trial demonstrated that heat-inactivated *Lactobacillus gasseri* CP2305 significantly improved gastrointestinal function, particularly in individuals with constipation tendencies. Subjects consuming CP2305-enriched fermented milk showed enhanced stool consistency and frequency [110]. Additionally, fecal analysis revealed increased concentrations of SCFAs and a significant rise in beneficial *Clostridium* cluster IV populations, indicating enhanced gut microbial activity [110]. These findings suggest that heat-inactivated CP2305 exerts regulatory effects on bowel function through modulation of gut microbiota and metabolite production. Further, Tarrerias et al. revealed that diarrhea-predominant irritable bowel syndrome significantly impairs quality of life, with high rates of fecal incontinence and poor tolerance to fiber and dairy in half of the patients [111]. Nutritional management should be integral to treatment. Inactivated *Lactobacillus LB* with fermented culture medium showed symptom relief without side effects, but controlled trials are needed to confirm efficacy.

**Figure 2:**
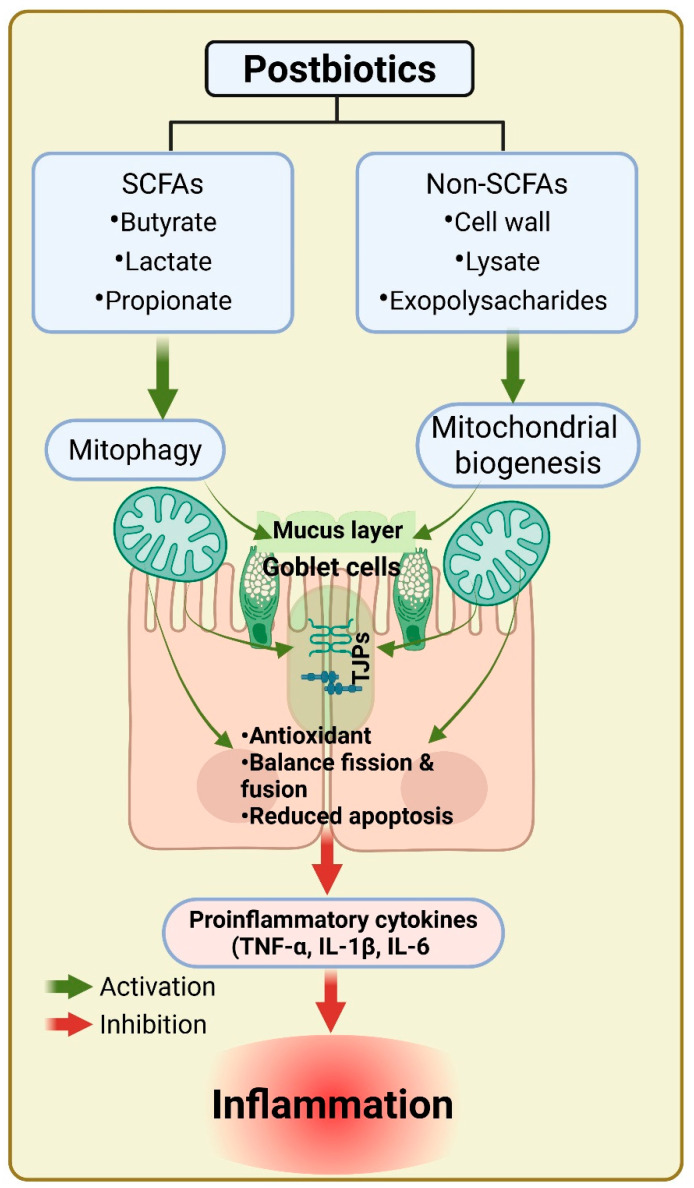
Postbiotics modulate intestinal homeostasis by targeting mitochondrial function and reducing inflammation. Short-chain fatty acids (SCFAs), including butyrate, lactate, and propionate, stimulate mitophagy, while non-SCFAs such as cell wall components, lysates, and exopolysaccharides promote mitochondrial biogenesis. These mitochondrial regulatory effects enhance goblet cell function, maintain the mucus layer and tight junctions (TJs), and support redox balance, mitochondrial dynamics, and cell survival. Collectively, these mechanisms suppress the production of proinflammatory cytokines (TNF-α, IL-1β, IL-6), thereby mitigating inflammation.

In summary, postbiotics not only support mitochondrial health through antioxidant and bioenergetic pathways but also modulate apoptosis, inflammation, and microbial balance, presenting a promising therapeutic approach for IBD (Figure 2).

## 6. Conclusions

One of the main causes of chronic inflammation and poor cellular energy metabolism in the development of IBD is mitochondrial dysfunction. Postbiotics restore mitochondrial function through mechanisms like lowering oxidative stress, increasing mitochondrial biogenesis, and modifying inflammatory pathways, and therefore they may offer patients with IBD alternative treatment options. Postbiotics may be a crucial component of IBD treatment, addressing the disease’s underlying metabolic and mitochondrial dysfunction.

Mitochondrial dysfunction is a critical factor in the pathogenesis of IBD, with aging exacerbating these effects. Understanding the mechanisms linking mitochondrial dysfunction, aging, and IBD can provide valuable insights for developing targeted therapies to improve outcomes in elderly patients with IBD. Future research should focus on elucidating these mechanisms and translating findings into clinical interventions.

## Figures and Tables

**Table 1:** Postbiotic-Based Interventions: Strain-Specific Effects and Therapeutic Outcomes Across Preclinical and Clinical Models of Gut Inflammation.

Strains	Postbiotics	Study Type	Therapeutic Outcomes	References
Atypical *Escherichia coli*	Butyrate	In vivo	Anti-inflammatory; improves gut integrity	[104]
*Bifidobacterium bifidum B1628*	Heat killed cells	In-vivo DSS-induced colitis model	Anti-inflammatory; increase beneficial gut microbiome	[105]
*Lactobacillus plantarum*	Heat-killed cells	DSS-induced colitis mice	Immunomodulator; increase tight junction proteins	[23,106]
*Limosilactobacillus fermentum HF06*	Heat-killed cells	DSS-induced colitis mice	Decrease intestinal barrier damage	[107]
*Lactobacillus*	Lipoteichoic acid	DSS-induced colitis model	Decrease proinflammatory and increase anti-inflammatory cytokines	[108]
*Lactobacillus paracasei TK1501*	Lipoteichoic acid and peptidoglycan	DSS-induced colitis mice	Ameliorate the mucin-2 expression and boost the phagocytosis	[109]
*Lactobacillus* spp.	Cell wall contents	Lipopolysaccharide-induced colitis rats	Mitigate immune-mediated inflammation and oxidative stress	[95]
*Lactobacillus gasseri* CP2305	Fermented milk-based beverage	Human	Increase the concentrations of short-chain fatty acids and the population of Clostridium in fecal matter	[110]
*Lactobacillus LB*	Inactivated fermented culture medium	Human	Reduce pain scores and improve quality of life in diarrhea-predominant irritable bowel syndrome	[111]
*Bifidobacterium bifidum MIMBb75*	Heat-inactivated	Human	Alleviates IBS and symptoms	[103]

## Data Availability

No new data were created or analyzed in this study.
